# Assessment of heat shock protein (HSP60, HSP72, HSP90, and HSC70) expression in cultured limbal stem cells following air lifting

**Published:** 2010-08-18

**Authors:** Marzeih Ebrahimi, Parvaneh Mohammadi, Arezoo Daryadel, Hossein Baharvand

**Affiliations:** 1 Department of Regenerative Medicine, Royan Institute for Stem Cell Biology and Technology, ACECR,Tehran, Iran; 2 Department of Stem Cells and Developmental Biology, Royan Institute for Stem Cell Biology and Technology, ACECR,Tehran, Iran; 3 Department of Developmental Biology, University of Science and Culture, ACECR, Tehran, Iran; 4 Department of Veterinary Physiology, University of Zurich, Zurich, Switzerland

## Abstract

**Objectives:**

The aim of this study is to create an ex vivo model to examine the expression of major heat-shock protein (HSP) families; HSP60, HSP72, and HSP90, and heat-shock cognate 70 (HCS70) at the mRNA and protein level in differentiating corneal cells from limbal stem cells (LSC) following air exposure.

**Methods:**

Limbal biopsies taken from cadaveric normal human limbus were cultivated as explants on human amniotic membrane (HAM) and plastic dish (PD). Corneal differentiation was induced by air lifting for 16 days. The expression of putative LSC markers (P63 and ATP-binding cassette G2 [ABCG2]), corneal markers (keratin 3 [K3/12] and connexin 43 [CX43]), and HSP60, HSP72, HSP90, and HSC70 were tested by RT–PCR, immunofluorescence, and flow cytometry pre- and post-air exposure. Fresh limbal and corneal tissues were used as control groups.

**Results:**

Air lifting induced corneal differentiation with a decrease in the number of P63^+^ cells and an increase in the number of K3^+^/CX43^+^ cells, which characterized transient amplifying cells (TACs). Moreover, denuded HAM provided a superior niche for LSC proliferation and phenotype maintenance in vitro. Additionally, we have evidence that expressions of HSC70 as well as HSP72 were enhanced through corneal differentiation and HSP90 post-air lifting in vitro and in vivo. HSP60, however, was not detected in either LSC or corneal cells, in vivo and in vitro.

**Conclusions:**

These results suggest that corneal differentiation following air exposure may regulate HSP72 and HSC70 expression. In addition, HSP72 and HSP90 may protect LSC and corneal cells against oxidative stress.

## Introduction

Heat-shock proteins (HSPs) are highly conserved proteins constitutively expressed in most cells under normal physiologic conditions whose expressions are induced by environmental stresses [1]. HSPs play an important role in embryonic development, cell cycle progression, cell differentiation, hormonal stimulation in vertebrate cells, and growth in microorganisms [2-5]. The first evidence of the presence and function of HSPs in the eye came from Barbe et al. [6] who have shown that induction of HSPs by hyperthermia correlated with the time when photoreceptors were protected from light-induced damage. In another attempt, expressions of HSP27, HSP70, and/or heat-shock cognate 70 (HSC70) were determined in many unstressed ocular tissues, including the retina and cornea [7,8]. Their expressions upregulated at wound sites which suggested their roles in ocular organogenesis and regeneration [9,10]. While it has been proposed that HSPs affect eye development in vivo, there is no model to directly show their roles in corneal differentiation. Thus, here we have developed an in vitro model to address two main questions. First, which HSPs are expressed through corneal differentiation? Second, what is the outcome of HSPs’ expression during air lifting; is it cell supportive of oxidative stress? To answer these questions limbal cells were cultivated on human amniotic membrane (HAM) and plastic dishes (PD), and exposed to air as inducers of corneal differentiation. Next HSPs, as well as limbal/corneal markers pre- and post-air lifting, were examined to gain a better understanding of the function of these proteins during corneal differentiation. HSP60, HSP72, HSP90 which have been categorized as inducible forms and HSC70 which has been reported as the structural form were selected for this study. All have been reported to express in cornea epithelium [10-13], however their functions remain unclear.

## Methods

This study was approved by the Institutional Review Board and Ethical Committee of Royan Institute (Tehran, Iran) and all experimental procedures were performed in accordance with the Declaration of Helsinki.

### Isolation and cultivating of limbal explants

Normal human eye globes (age averaged=43.5 n=18) were obtained from the Central Eye Bank of Iran (Tehran, Iran). They were preserved for less than 24 h post mortem. Isolation and cultivation of limbal biopsies were done as previously reported [14]. Briefly, under surgical microscopy; the central cornea, excess sclera, conjunctiva and iris were carefully removed, and next the remaining tissues were treated with 10 µg/ml dispase II (17105–041; Gibco, Aukland, NZ) in HBSS (14185; Gibco) for 15 min at 37 °C under humidified 5% CO_2_ to facilitate isolation of stroma and limbal endothelium. Each remaining ring was then divided into 1×1 mm^2^ segments. One piece of the segment was placed epithelial side up at the center of PD and HAM which had been denuded by 0.05% trypsin/EDTA at 37 °C for 5 min. The explants were cultured in DMEM/Ham's F-12 (1:1) supplemented with 10% FBS, 0.5% dimethyl sulphoxide (DMSO; D2650; Sigma, Steinheim, Germany), 2 μg/ml epidermal growth factor (EGF; E9644; Sigma), 5 μg/ml insulin (57590; Sigma), transferrin (T-1147; Sigma), sodium selenite (556, Sigma), 0.5 μg/ml hydrocortisone (H0888–56; Sigma), and 50 μg/ml penicillin/streptomycin. Cultures were incubated in a humidified incubator in 95% air and 5% CO_2_ for 14 days with approximately 4 ml medium that was replaced every three days. Corneal differentiation was induced by decreasing the volume of culture medium to 800 μl and exposing cells to the air for 16 days. Medium was semi-depleted every other day.

### Immunoflourescence staining for detection of specific epithelial markers and HSPs

Immunofluorescent staining was performed by conventional methods. Briefly, cornea and limbal tissues, and epithelial outgrowth were fixed with 4% freshly buffered paraformaldehyde (pH 7.4) at 4 °C for 10 min. Slide mounted sections were deparaffinized, rehydrated and washed with distilled water, followed by antigen retrieval with 20 mM Tris solution. Slides, as well as fixed cells, were permeabilized with 0.2% Triton X-100 for 10 min and pre-incubated with 10% goat serum for 45 min followed by incubation with primary antibodies in their respective dilutions: anti-p63 (1:100, MAB4135; Chemicon, Temecula, CA), anti-CX43 (1:100, CX43; C8093; Sigma), anti-K3/K12 (1:1,000, C9097–34; US Biological, Swanpscott, MA), anti-HSP60 (1:100, SPA-807E; Stressgen, San Diego, CA), anti-HSP72 (1:100, SPA-815, Stressgen), and anti-HSP90 (1:100, SPE-835, Stressgen). Then, samples were washed with PBS and incubated with fluorescence isothiocyanate (FITC)-conjugated anti-mouse IgG (F9006; Sigma), anti-mouse IgM for CX43 (F9259; Sigma), anti-rat IgG (F 6258; Sigma) in a 1:200 dilution for 60 min at room temperature. Nuclei were counterstained with 5 μg/ml DAPI (4', 6-diamidino-2-phenylindole) or propidium iodide (PI-P4170; 10μg/ml; Sigma) and analyzed by fluorescent microscopy (Nikon, Tokyo, Japan). The percentage of positive cells was calculated by counting cells in 10–15 random fields of view. All assays were performed in triplicate.

### Light-scattering measurements and flow cytometry

Cells were measured by flow cytometry to achieve better understanding of their morphology, size and granularity. In this context forward side scatter (FSC) provides information about size whereas side scatter (SSC) represents an indirect measure of the cell granularity within a particle [15]. Therefore cells were categorized into three different areas on SSC as previously mentioned [15]; R1, 10–200 or limbal stem cell (LSC); R2, 200–400 or transient amplifying cells (TAC); and R3, >400 or terminal differentiated cells (TDC).

Cell suspensions of cultured limbal epithelial cells at days 14 and 30 (before and after air lifting) were removed for light-scattering measurement and flow cytometric analysis. A cell count was performed and adjusted to approximately 5×10^4^ cells. The light-scattering properties of the cells were measured using an Argon laser (488 nm) as the probing beam. Red light emission was simultaneously measured to any dead, PI-stained cells seen during analysis. For flow cytometry, cells were fixed and permeabilized with ice-cold methanol for 10 min and then blocked by 10% goat serum. Then, cells were incubated with anti-HSP90, anti-HSC70, and anti-HSP72 for 1 h at 4 °C. Finally, attached antibodies were detected by FITC-conjugated anti-mouse IgG and anti-Rat IgG for 45 min. The labeled cells were analyzed using a FACScan flow cytometer (Becton-Dickinson, San Jose, CA).

### Reverse transcription-polymerase chain reaction (RT–PCR)

Total RNA was isolated from cultivated limbal epithelial cells pre/post air lifting (days 14 and 30) using trizol (155 g6–018; Invitrogen, Carlsbad, CA). Prior to RT, RNA samples were digested with 1 U/μl DNase I (EN0521**;** Fermentas, Leon-Rot Germany**)** to remove contaminating genomic DNA. Standard RT was performed using 2 μg total RNA, 0.5 μg oligo (dT) 18 per reaction and RevertAidTM First Strand cDNA Synthesis kit (K1622; Fermentas) according to the manufacturer's instructions. Reaction mixtures for PCR included: 2 μl cDNA, 1× PCR buffer (AMSTM; CinnaGen Co., Tehran, Iran), 200 μM dNTPs, 0.5 μM of each antisense and sense primers and 1U Taq DNA polymerase. The PCR primers and annealing temperature of the amplified products are presented in Table 1. PCR reactions were done on a Mastercycler gradient machine (Eppendorf, Hamburg, Germany). PCR was performed at 94 °C for 5 min, 94 °C for 45 s, 59 °C for 45 s and 72 °C for 30 s; for 35 cycles. Products were electrophoresed on 2% agarose gel, stained by ethidium bromide (0.5 μg/ml) and photographed on a UV transilluminator (Uvidoc, Cambridge, UK).

**Table 1:** Human primers and conditions.

**Gene**	**Primer sequences (5′-3′)**	**Annealing temperature (°C)**	**Product size (bp)**
*ABCG2*	F: 5′-CTCTTCTTCCTGACGACCAACC-3′	58	515
	R: 5′-CACACTCTGACCTGCTGCTAT G-3′		
*HSP90β*	F: 5′-AGGTGGACTGTTTCCTCTCTC-3′	58	341
	R: 5′-TTGCTCACTTGCTTGCTTGTTG-3′		
*KRT3*	F: 5′-AGACTTCAAGAAGAAATATGA G-3′	60	141
	R: 5′-TCATCTATCAAGGCATCCAC-3′		
*HSPA8*	F: 5′-TCTTGGCACCACCTACTCTTG-3′	58	125
	R: 5′-CATCACCGATCAACCGTTCAG-3′		
*HSPD1*	F: 5′-CGCTGAAGATGTTGATGGAG-3′	60	107
	R: 5′-TTTCTATTGTCACCAAACCCTG-3′		
*HSP90α*	F: 5′-GAGATCAAAGACTACAGTCCC T-3′	58	150
	R: 5′-GTTCGTGCTCATACTTGGTC-3′		
*P63*	F: 5′-GAGGTTGGGCTGTTCATCAT-3′	58–62	229
	R: 5′-GTGGGAAAGAGATGGTCTGG-3′		

In the table, HSPA8 indicates HSP70 & HSC70 and HSPD1 indicates HSP60.

### Statistical analysis

The percentages of positive cells were compared using paired samples *t*-test in each group and by independent *t*-test between groups by SPSS software (SPSS, Chicago, IL). Results were expressed as the mean±SD and p<0.05 was considered to be statistically significant.

## Results

### Morphological properties of cultivated LSC following air exposure

**Figure 1:**
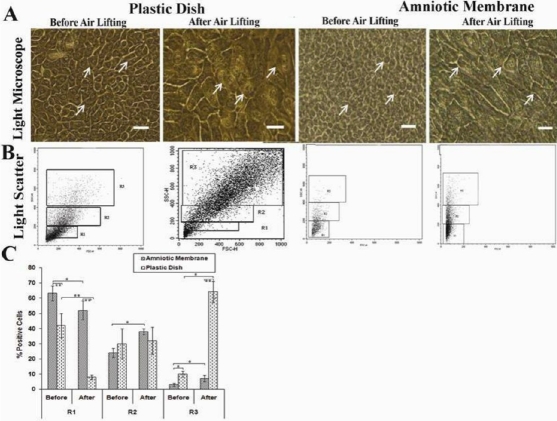
Human limbal explants expanded on human amniotic membrane (HAM) and plastic dish (PD) for 14 days, followed by air lifting for 16 days. **A**: Phase-contrast microscopy of the cells shows that cells on HAM are small and compact compared with those in PD pre air lifting; however, the morphology of the cells in the PD group changes to squamous epithelial cells post air lifting. Scale bar: 100 µm. **B**: Assessment of cell heterogeneity in R1: 10–200 or limbal stem cell (LSC), R2: 200–400 or Transient amplifying cells (TAC), and R3: >400 or terminal differentiated cells (TDC) by light scatter showed that cells in PD are more differentiated than those in AM. **C**: The comparison of cells in PD and AM groups before and after air lifting shows cells differentiated to large cells with high granularity. Results were presented as percentage of 3 different experiments and expressed as mean±SD. The astereisk indicates a p<0.05 and the double asterisk indicates a p<0.01.

Epithelial cells from explants began to migrate on a cellular HAM or PD, forming a rim around the limbal fragment within 2–5 days (2.26±1.58 days on denuded HAM and 4.12± 1.23 days on PD). The epithelial sheets grown from the limbal explants on denuded HAM were compact, small and uniform with a nuclear to cytoplasm ratio (N/C) of approximately 1:1 before air lifting. (Figure 1A). In contrast, expanded cells on PD increased in size with polygonal borders, and showed discontinuous cell expansions with irregular leading edges and a small N/C (Figure 1A). Numerous cells in the PD group and some in the HAM group exhibited changes in their size, shape and N/C with the embossed nucleus after air lifting. The majority of cells in the PD group differentiated to cuboid wing cells, flat squamous cells and more spread out-looking cells when compared to HAM, which had some polygonal and basal column shaped cells distributed among other round cells (Figure 1A).

### Light scattering of the cells

Forward scatter (FSC), which serves as an indirect measure of overall cell size, and side scatter (SSC), which represents an indirect measure of the cell granularity, were used to evaluate the light-scattering properties of cells pre- and post-air lifting. We found that the size and granularity of cells cultured on PD increased after air lifting, but only the granularity of cells increased in the HAM group (Figure 1B). By categorizing cells into three different areas on SSC as previously mentioned [15]; we found that cells cultivated on PD were more heterogenic (approximately 42% LSC, 30% TAC, 10% TDC) than HAM (approximately 63% LSC, 34% TAC, <5% TDC; Figure 1C). Most noteworthy was that air lifting decreased cells in the LSC area whereas cells increased in the TAC and TDC areas (Figure 1C), which was dramatically high in the PD group (Figure 1C). These results imply that air exposure promotes corneal differentiation in vitro.

### Air lifting increases the expression of corneal differentiation markers (K3 and CX43) in cultivated LSCs

**Figure 2:**
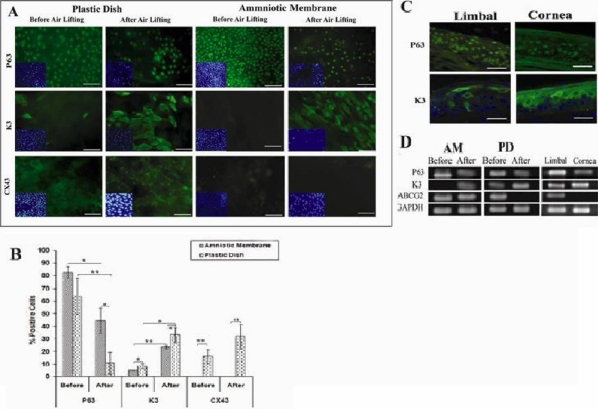
Evaluation of limbal stem cell and corneal differentiation markers pre and post air lifting in cultured limbal cells on AM and PD groups. **A**: Representative fluorescent staining profile shows that P63, a putative stemness marker, expressed in nucleus and K3, a differentiation marker, in cytoplasm and Cx43 in cell membrane and near the nucleus in cultured cells on amniotic membrane (AM) and plastic dish (PD) before and after air lifting. The nuclei were stained by Dappi (Blue). Scale bar: 100 µm. **B**: Comparative data for PD and AM group for P63, K3, and CX43, before and after air lifting by flowcytometry analysis. **C**: Immuno staining of cornea and limbal tissues for P63 and K3 Marker. Scale bar: 100 µm. **D**: RT–PCR shows the gene expression of P63,K3, ABCG2 and GAPDH as internal control before and after air lifting in AM, PD as well as limbal and corneal cells. Results were presented as percentage of 3 to 4 different experiments and expressed as mean±SD. The asterisk indicates a p<0.05 and the double asterisk indicates a p<0.01.

We sought to determine whether exposure to the air could influence the phenotype and differential gene expression of cells cultured on HAM and PD as mentioned in the Methods. P63, a transcription factor that has been reported to be a specific marker for LSCs [16,17], was expressed in the nuclei (Figure 2A) and basal layer of the cornea (close to base membrane) and whole thickness of the limbal area (Figurer 2C). Furthermore, 82.5%±4.5% and 63.8%±14.19% of cells in HAM and PD outgrowing cells, respectively, were positive for P63 pre-air lifting (Figure 2B). However, they were significantly reduced post-air lifting (44.4%±9.98% in HAM versus 10.81%±8.7% in PD; Figure 2B, p<0.01). RT–PCR confirmed immunostaining results in vivo and in vitro (Figure 2D). Additionally, the expression of *ABCG2*, a putative LSC marker that was expressed in the cytoplasm of LSCs [16,17], down-regulated dramatically or was not detected in cells cultured on PD or in corneal tissue post-air lifting (Figure 2D); however, it was expressed in cells cultivated on HAM and limbal tissue pre- and post-air lifting (Figure 2D).

For estimating the cornea differentiation, connexin 43 (CX43) and K3 [16] were chosen as corneal specific markers. Positive CX43 staining was punctuated in appearance and confined to the cell membrane of adjacent cells (Figure 2A), compatible with its known function in the formation of gap-junction channels. It was also observed to be in close proximity to the nucleus (Figure 2A). Most obviously, 5.34%±0.12% of cells in HAM and 8.45%±1.32% in PD were positive for K3 pre-air lifting (Figure 2B), which significantly increased to 23.5%±1.3% and 33.18%±5.8% in HAM and PD, respectively, post-air exposure (Figure 2B, p<0.01). K3 was detected in the cytoplasm of superficial cells of the cornea and in a few superficial cells of the limbus (Figure 2C) as well as the superficial layer of cultured cells (Figure 2A). The percentage of positive cells following air lifting increased significantly in both group**s** and was higher in the PD group (p<0.05, Figure 2B).

Similarly, RT–PCR confirmed results from immunostaining in vitro; however, expression of *K3* was detected in limbal as well as corneal epithelium (Figure 2D) due to the existence of TAC in limbal biopsies.

Taken together, air exposure caused a decrease in the number of P63^+^ cells, about 5.9 and 1.86 fold, in the PD and HAM groups, respectively. Conversely, the percentage of K3^+^ cells were enhanced about fourfold in both groups, and CX43 was enhanced approximately twofold only in the PD group (Figure 2B). Thus exposure to air promotes corneal differentiation which is dependent on extra-cellular matrices.

### Expression of HSP72, HSC70, and HSP090 are induced following corneal differentiation

**Figure 3:**
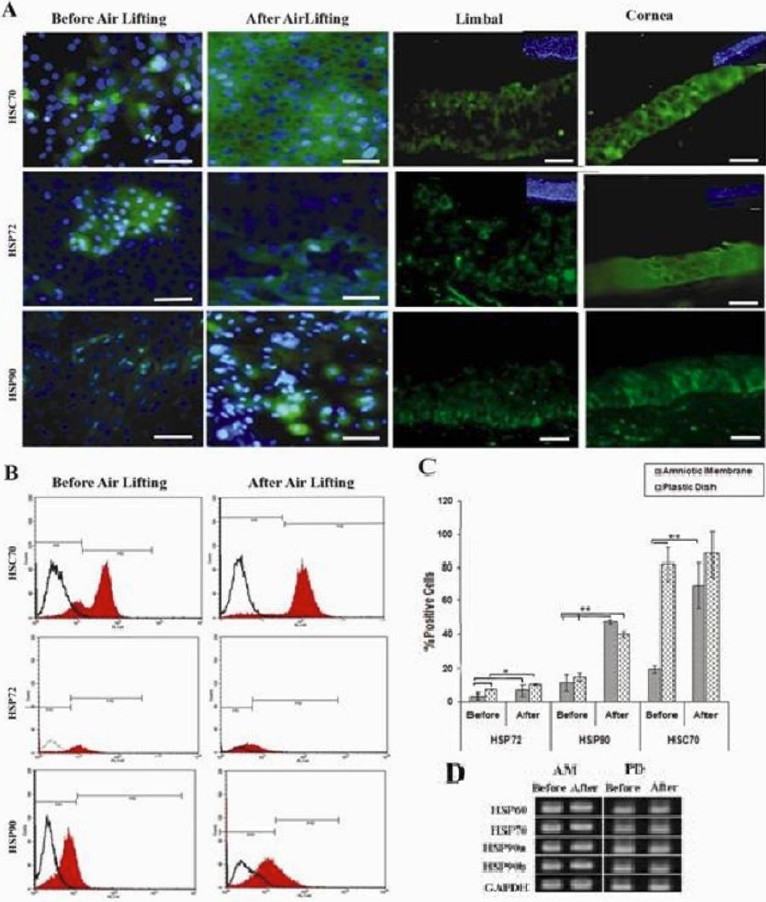
Evaluation of HSPs pre and post air lifting in AM and PD groups. **A**: Representative fluorescent staining indicates HSC70, HSP72, and HSP90 are expressed in cytoplasm. HSP72 also detected in few nuclei of cells cultured on amniotic membrane before and after air lifting. The nuclei were stained by DAPI (Blue). Scale bar: 100 µm. **B**: Representative histograms show the percentage of cells positive for HSC70, HSP72, and HSP90 on plastic dish. Black line indicates isotype control and red points out HSPs. **C**: Comparative data for PD and AM groups for HSPs before and after air lifting. **D**: RT–PCR shows the gene expression of HSC70, HSP72, and HSP90 before and after air lifting in AM and PD. GAPDH was used as internal control. Results were presented as percentage of 3 to 4 different experiments and expressed as mean±SD. The asterisk indicates a p<0.05 and the double asterisk indicates a p<0.01.

This study was performed to determine which HSPs may be regulated by corneal differentiation. Our results clearly revealed that both HSC70 and HSP72 were present in the cytoplasm and nuclei when assessed by immuno-cytochemistry (Figure 3A). It is interesting to note that HSP72, HSC70, and HSP90 did not follow a similar pattern of expression, which may suggest the specific roles that each could have in the biology of the cornea. HSC70 had been localized in the culture margin in which high amounts of differentiated cells were observed. Notably, in vivo studies showed few cells in the superficial layer of limbal epithelium that expressed HSP72 and HSC70, but all layers of the cornea were positive for these stress proteins (Figure 3A). Quantified results by flow cytometry (Figure 3B) showed that 81.90%±19.40% of cells were positive for HSC70 and 6.73%±0.16% were positive for HSP72 on PD when compared to 10.54%±7.54% and 3.09%±1.33% on HAM, respectively (Figure 3C). Furthermore, expression of HSP72 and HSC70 enhanced up to 2.12 and 3.51 fold, in the HAM group after air lifting (p<0.05, Figure 3C).

To evaluate the influence of air lifting on LSCs, we compared both groups pre- and post-air lifting and found that expression of HSP90 increased dramatically in the HAM group (about 5.49 fold, p=0.01, Figure 3C). The number of HSP90^+^ cells increased by 2.80 fold in the PD group, but the difference was not significant (p=0.2, Figure 3C). HSP90 was detected in the cytoplasm and in the nucleus (Figure 3A) of cultivated cells, and the basal cells of limbal and corneal epithelium (Figure 3A). Among HSPs, only expression of HSP60 was absent in corneal, limbal epithelium and cultivated cells at the protein level, however it was expressed strongly at the mRNA level as well as other HSPs (HSP72, HSP90, and HSC70) pre- and post-air lifting (Figure 3D), which may correlate with regulatory function of HSPs at the mRNA level.

## Discussion

### Amniotic membrane promotes proliferation and limits differentiation of LSCs in culture

In this study we have shown that cultured cells on HAM before air lifting are small, with low granularity and high in N/C. They are P63^2+^/K3^–^/CX4^–^, which characterized limbal basal cells [15,18,19]. However, cells on PD are large, heterogeneous and have a low N/C with high granularity and are P63^+-^/K3^+-^/CX4^–^ before air lifting, which characterized TAC at various differentiation steps [16,17]. Cells in the superficial layer of the cornea and some cells in the PD group, after air exposure, expressed K3 and CX43 which characterized terminate differentiated cells (TDC). Therefore we assumed that cells not only differentiate faster on plastic than on HAM but they also entered the proliferation stage a little later [20], which confirmed our previous observations that denuded HAM provides a superior niche for limbal SC proliferation and phenotype maintenance in vitro [14,21,22]. It has been suggested that proteins such as lumican, osteoglycin/memican, collagen type α1 (IV) chain IV and fibrinogen periplakin; pidogen 2, transglutaminase 2 (TG2) and tubulointerstitial integrin α6 [21]; laminin-1,-5; and fibronectin, which are abundant in amniotic membrane, promote epithelial adhesion and migration [23].

### Air exposure induces corneal differentiation in vitro

Our next step determined the phenotype and gene expression of putative LSC and corneal differentiation markers following air exposure. Cellular morphology in both groups after air lifting changed to squamous structures with high granularity and cell size, with a reduction of P63 expression, and K3 and CX43 induction which characterized TAC [16,20]. Moreover, cells cultivated on PD were heterogeneous and contained both late TAC and TD cells after air lifting. However, these results should be confirmed by dual or triple staining of cells. Interestingly, CX43 was not expressed in cells cultured on AM which confirmed the earlier notion of AM in limbal cells’ survival. Finally, it is tempting to speculate that corneal differentiation cannot be completed on AM in vitro and needs to be completed in vivo.

### Expression pattern of HSPs during corneal differentiation by air lifting

Although many studies have confirmed the role of HSPs in embryonic development and organogenesis [2,4] there is no evidence as to their role in the case of corneal differentiation or development. Here, for the first time we have used LSC culture to investigate the regulation of the inducible forms of HSPs (HSP60, HSP72, and HSP90) and structural form (HSC70) during corneal differentiation.

Our result determined that expression of HSP72 (inducible form of HSP70) and HSC70 increase 2.18 and 7.17 fold, respectively in cultured cells on PD compared to HAM pre- air exposure. Since cells on PD were more differentiated before air lifting, and as a result of increased HSC70 and HSP72 following air exposure (6.5 and 2.12 fold, respectively) in HAM, we believed that induced expression of these two isoforms of HSPs are associated with corneal differentiation. Unlike the stress-induced proteins HSC70, however, may not provide significant protection against stress-induced pathologies as it did not increase in the PD group after air lifting. Most intriguing was the observation of HSC70 expression in 80% of PD cells before air lifting, which did not change after air exposure; therefore, we assumed it expressed at early TACs and not late TACs or TD cells, which are confirmed by in vivo studies [24]. HSC70 can interact with various co-chaperone proteins and guide the sequential restructuring of stable or transient protein complexes to promote a temporal and spatial regulation of the endo- and exocytotic machinery [25]. Yet, more studies using siRNA or neutralizing antibodies for diminished function are needed to clear the molecular mechanism of HSP72 and HSC70 action in corneal differentiation and HSC70 in TACs.

In addition, here we speculate that HSP72 expresses in early/late TAC as well as TD cells because of its enhancement in post-air lifting in both groups. Also HSP72 localization was observed in the cytoplasm of basal and superficial cells (few cells) in the limbus and full thickness of the cornea in vivo, which confirmed its role in proliferation, differentiation and migration in the cornea [12] and may be the restoration of the intracellular structure that is critical for cell survival following air exposure [26,27]. HSP72 assists in the folding of newly synthesized polypeptides, assembling of multiprotein complexes and the transporting of proteins across cellular membranes in an ATP-dependant way [28]. Moreover, it can effectively inhibit cellular death processes, apoptosis or necrosis, and thereby increase the survival of cells exposed to a wide range of lethal stimuli [28].

Furthermore the results presented here show that expression of HSP90 increases during air exposure in both groups, and there is no significant difference between cells on HAM and PD pre-air exposure to express this marker. However post-air exposure cells on HAM express a high level of HSP90 compared to the PD group. In vivo studies reveal that the expression of HSP90 is limited to basal cells of limbal, basal and wing cells of the cornea. Thus, it seems to have an important role in cell protection, especially in highly proliferated cells and not terminally differentiated cells. HSP90 is involved in the cell cycle and is a regulator of the growth and differentiation processes during postnatal development of the rat brain and kidney [13]. Also we have suggested that amniotic membrane protects cells with increasing HSP90 expression against oxidative stress or environmental stress, as mentioned previously [17,24].

Finally, expression of HSP60 as well as other HSPs (HSP72, HSC70, and HSP90) at the mRNA level pre- and post-air exposure strongly supports this concept that HSPs at the mRNA level play an important role in the regulation of cell function and cell signaling as well as ocular organogenesis [29]; however this must be confirmed by functional analysis.

In conclusion, our study, for first time, provides primary evidence that HSP72 and HSC70 proteins are components of the corneal differentiation program; the corneal epithelial cells, like other cells, respond to environmental stress by increasing HSP70 and HSP90; and the abundance of HSC70 in corneal and limbal epithelial cells in vivo and in vitro correlates with the proliferative capacity of undifferentiated cells.
